# Vampires and nurses are rated differently by younger and older adults—Age-comparative norms of imageability and emotionality for about 2500 German nouns

**DOI:** 10.3758/s13428-019-01294-2

**Published:** 2020-02-12

**Authors:** Thomas H. Grandy, Ulman Lindenberger, Florian Schmiedek

**Affiliations:** 1 Center for Lifespan Psychology, Max Planck Institute for Human Development, Berlin, Germany; 2 Working Group on Cardiovascular Magnetic Resonance, Experimental and Clinical Research Center—a joint cooperation between the Charité Medical Faculty and the Max Delbrück Center for Molecular Medicine and HELIOS Hospital Berlin-Buch, Department of Cardiology and Nephrology, Berlin, Germany; 3 DIPF | Leibniz Institute for Research and Information in Education, Frankfurt am Main, Germany

**Keywords:** Imageability, Imagery, Emotionality, Valence, Age differences

## Abstract

Imageability and emotionality ratings for 2592 German nouns (3–10 letters, one to three phonological syllables) were obtained from younger adults (21–31 years) and older adults (70–86 years). Valid ratings were obtained on average from 20 younger and 23 older adults per word for imageability, and from 18 younger and 19 older adults per word for emotionality. The internal consistency (Cronbach’s *α*) and retest rank-order stability of the ratings were high for both age groups (*α* and *r* ≥ .97). Also, the validity of our ratings was found to be high, as compared to previously published ratings (*r* ≥ .86). The ratings showed substantial rank-order stability across younger and older adults (imageability, *r* = .94; emotionality, *r* = .85). At the same time, systematic differences between age groups were found in the mean levels of ratings (imageability, *d* = 0.38; emotionality, *d* = 0.20) and in the extent to which the rating scales were used (imageability, *SD* = 24 vs. 19, scale of 0 to 100; emotionality, *SD* = 26 vs. 31, scale of −100 to 100). At the descriptive level, our data hint at systematically different evaluations of semantic categories regarding imageability and emotionality across younger and older adults. Given that imageability and emotionality have been reported, for instance, as important determinants for the recognition and recall of words, our findings highlight the importance of considering age-specific information in age-comparative cognitive (neuroscience) experimental studies using word materials. The age-specific imageability and emotionality ratings for the 2592 German nouns can be found in the electronic supplementary material 1.

**Electronic supplementary material:**

The online version of this article (10.3758/s13428-019-01294-2) contains supplementary material, which is available to authorized users.

The present study provides age-comparative ratings for 2592 German nouns for *imageability*—as the capacity to evoke perceptual or mental images—and *emotionality*—as the capacity to elicit pleasant or unpleasant and awkward feelings or emotions—from younger (21–31 years old) and older (70–86 years old) adults. The imageability and emotionality of words have been found to be important determinants for their recognition and recall in memory experiments (e.g., Kensinger, Brierley, Medford, Growdon, & Corkin, 2002; Paivio, Yuille, & Rogers, 1969; Rubin & Friendly, 1986). Moreover, age differences between younger and older adults in imageability and emotionality ratings have been reported (e.g., Grühn & Smith, 2008; Kensinger, Brierley, Medford, Growdon, & Corkin, 2002), thus implicating these word characteristics as a potential confound in age-comparative memory experiments. Nonetheless, despite the prominent role of memory as a topic in research on cognitive aging and the cognitive neuroscience of aging (e.g., Brod, Werkle-Bergner, & Shing, 2013; Lindenberger, 2014; Shing, Werkle-Bergner, Brehmer, Müller, Li, & Lindenberger, 2010), so far age-specific information regarding imageability and emotionality of words has been lacking for a larger body of German nouns (Kanske & Kotz, 2010; Lahl, Göritz, Pietrowsky, & Rosenberg, 2009; Schmidtke, Schröder, Jacobs, & Conrad, 2014; Võ et al., 2009; Võ, Jacobs, & Conrad, 2006; see also Hager & Hasselhorn, 1994a). The primary goal of the present study was to provide researchers with age-specific information for a large body of German nouns to control for or rule out differences in memory performance that may be attributed to differences in imageability and emotionality of presented words. Word ratings presented here have been implemented in a large-scale cognitive training study (Schmiedek, Lövdén, & Lindenberger, 2010).

Imageability (or imagery) as a psychological construct was introduced to experimental psychology by Paivio (1965) and has been ever since in the focus of memory research. On the one hand, imageability of words has been found to influence recall and recognition performance (e.g., Cortese, Khanna, & Hacker, 2010; Cortese, McCarty, & Schock, 2015; Paivio et al., 1969; Rubin & Friendly, 1986). On the other hand, imagery instructions—that is, the instruction to encode the presented words (or word pairs) as vivid images—are commonly employed in memory tasks (e.g., Bower, 1970; Paivio, 1971; Richardson, 1998; Shing, Werkle-Bergner, Li, & Lindenberger, 2008). Imageability ratings for words have been found to differ across different age groups (e.g., Emmerich, 1979; Forisha, 1975; Grühn & Smith, 2008). Consequently, as imageability plays an important explicit (by instruction) as well as implicit (by moderating performance) role in memory experiments, identification of words that differ considerably in their imageability across age groups is of high relevance.

The emotionality of stimuli has equally been reported to enhance their recall and recognition (Adelman & Estes, 2013; Hamann, 2001; Hamann, Cahill, & Squire, 1997; Kensinger et al., 2002; Rubin & Friendly, 1986). Grühn and Smith (2008) reported reliable differences in emotionality ratings between younger and older adults for a considerable number of adjectives. Moreover, several studies reported an influence of the emotionality of stimuli on age-related differences in memory (Charles, Mather, & Carstensen, 2003; Grühn, Scheibe, & Baltes, 2007; Grühn, Smith, & Baltes, 2005; Kensinger et al., 2002). Notably, findings reported by Kensinger and colleagues suggest that age-related differences in the recall of emotional words may be accounted for by age-related differences in the ratings of words as emotionally neutral, positive, or negative (see also Grühn & Smith, 2008). Consequently, the emotionality of words is an important property to control for in age-comparative cognitive (neuroscience) experiments.

The primary goal of the present study was to fill in the gap of age-specific imageability and emotionality norms for a larger body of German nouns as experimental control over known confounding factors is important to unambiguously understand changes in memory processes and the memory system across the lifespan. Nonetheless, the information provided may also be employed beyond memory research in the selection, control, manipulation, or analysis of stimulus characteristics in other experimental cognitive (neuroscience) settings (cf. Balota, Cortese, Sergent-Marshall, Spieler, & Yap, 2004; Cortese & Fugett, 2004).

Of note, the instructions for the word-rating procedure closely followed the recommendations in Hager and Hasselhorn (1994a; see also Cortese & Fugett, 2004; Toglia & Battig, 1978). Imageability and emotionality ratings for 725 and 556 words, respectively, out of the 2592 words were reported in Hager and Hasselhorn (1994b; see also Baschek, Bredenkamp, Oehrle, & Wippich, 1977; Offe, Anneken, & Kessler, 1981; Schwibbe, Räder, Schwitte, Borchardt, & Geiken-Pophanken, 1981), thus allowing us to assess the validity of the present word ratings in comparison to previously reported German word norms.

## Method

### Participants

The final sample comprised 29 younger (*M*_age_ = 26.2 years, *SD* = 2.3, range = 21–31 years; 15 women, 14 men) and 32 older (*M*_age_ = 75.4 years, *SD* = 3.5, range = 70–86 years; 17 women, 15 men) adults for imageability ratings, and 26 younger (*M*_age_ = 26.2 years, *SD* = 2.8, range = 21–31 years; 13 women, 13 men) and 25 older (*M*_age_ = 75.0 years, *SD* = 3.9, range = 70–86 years; 13 women, 12 men) adults for emotionality ratings. The participants were recruited from the participant pool of the Max Planck Institute for Human Development, Berlin, Germany (MPIB). All participants gave written informed consent according to institutional guidelines of the ethics committee of the MPIB and were paid for their participation (€10 per hour).

**Fig. 1:**
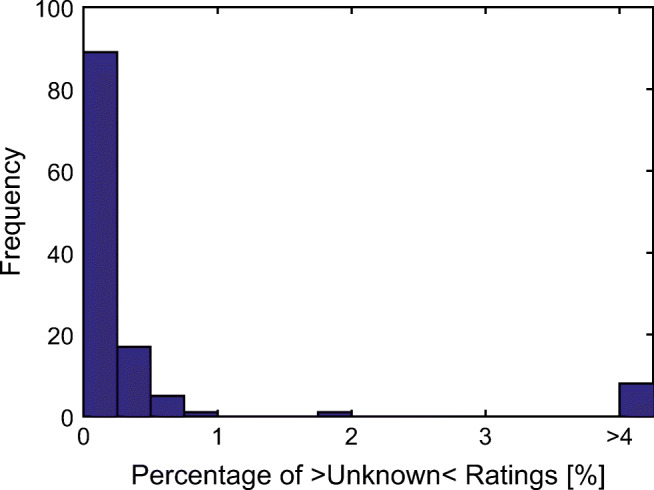
Frequency of relative occurrences of words rated as “unknown”, across age groups and imageability and emotionality ratings.

Participants who indicated that more than 1% of the rated words were unknown were excluded from further analyses (for the imageability ratings, three younger and one older adults were excluded; for the emotionality ratings, two younger and three older adults were excluded; see Fig. 1). For the final sample, the percentage of words rated as unknown amounted to 0.14% ± 0.21% for the imageability ratings (range = 0.0% to 0.9%), and 0.10% ± 0.16% (range = 0.0% to 0.6%) for the emotionality ratings, across both age groups.

### Word corpus

In all, 2592 German nouns with two to ten letters and one to three phonological syllables (one noun with four phonological syllables) were rated with respect to imageability and emotionality. The overall word corpus was compiled from existing German word corpora and previous studies conducted at the MPIB (Baayen, Piepenbrock, & Gulikers, 1995; Hager & Hasselhorn, 1994b; Shing et al., 2008; Singer, Lindenberger, & Baltes, 2003). In addition, 261 high-frequency words from the CELEX lexical database (Baayen et al., 1995) were added. All but 40 words are contained in the CELEX lexical database.

### Procedure

Participants took part in up to three 2-h sessions for the imageability and emotionality ratings, separately. The data were collected in 2005. For imageability ratings, the younger adults participated on average in 1.8 ± 0.9 (*SD*) sessions, rating on average 1824 ± 824 words, and the older adults took part in 2.3 ± 0.9 sessions, rating on average 1887 ± 790 words. For emotionality ratings, the younger adults participated on average in 1.7 ± 0.8 sessions, rating on average 1857 ± 770 words, and the older adults took part in 2.2 ± 0.8 sessions rating on average 2014 ± 737 words.

Participants were tested in groups of up to 20 in a large room. Word rating was conducted self-paced on personal computers. Participants were instructed with a standardized PowerPoint presentation regarding the general procedure and the criteria by which the nouns had to be rated prior to the word rating (see below). In addition, every participant obtained a printout of the instructions (see the Appendix) and could ask for assistance at any time during the session. To ensure equivalent anchoring of the ratings, a printed-out list of 25 words was provided, and the rating in each session started with these 25 anchor words (see the Appendix). For these 25 words, the ratings of the first session are reported in the word norm table. Moreover, the aggregated ratings of these 25 words for the first session served to estimate the consistency (Cronbach’s *α*; Cronbach, 1951), and for the first and second sessions, to estimate the reliability (rank-order stability), of the aggregate ratings.

The 25 anchor words and the remaining 2567 words were presented in random order. To ensure comparable numbers of ratings for every word, the number of ratings obtained per word was monitored online, and the words with fewer ratings were presented with higher probability. The average number of valid imageability ratings per word was 20.1 ± 1.3 (*SD*; range = 14 to 29) in younger adults, and 22.9 ± 1.0 (range = 20 to 32) in older adults. For the emotionality ratings, the average number of valid ratings per word was 18.4 ± 1.0 (range = 9 to 26) in younger, and 19.1 ± 0.7 (range = 8 to 25) in older, adults.

**Fig. 2:**
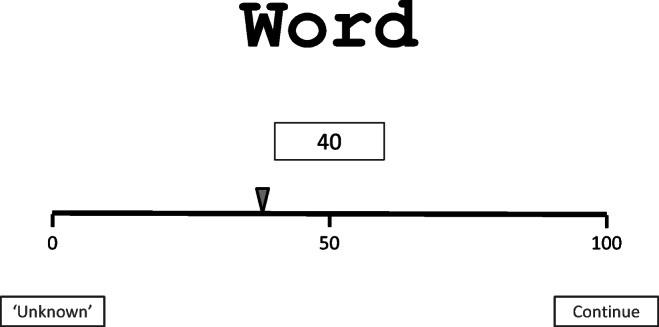
Layout of the presentation of a word and the rating scale on the computer screen.

The words to be rated were presented in the middle of the computer screen. A bar together with a pointer was presented below the presented word. The bar was marked at the ends and the midpoint with the respective scale values—that is, 0, 50, and 100 for the imageability ratings, and − 100, 0, and 100 for the emotionality ratings (see Fig. 2). Participants could either click with the cursor of their computer mouse at a location on the scale where they intended to place their rating, or pull the pointer along with their mouse cursor. A field above the scale indicated the specific numeric value that participants had assigned to the presented word. If participants were fine with their rating, they could click on the “continue” button. If a presented word was highly unfamiliar or was unknown to participants, they were instructed to indicate this by clicking with the cursor on the “unknown” button.

For *imageability* ratings, participants were instructed to rate the nouns regarding their capacity to evoke perceptual or mental images (cf. Paivio, Yuille, & Madigan, 1968; Paivio et al., 1969) on a scale from 0 to 100. Specifically, they were instructed to assign high numeric values if the presented word elicited perceptual or mental images promptly and easily, and to assign low numeric values if the presented word elicited perceptual images only slowly and with difficulty; if words generated perceptual or mental images neither easily nor with difficulty, participants were instructed to assign values around the middle of the scale. Furthermore, they were explicitly advised only to rate the presented word, and not to include associated perceptual or mental images that were evoked by the presented word (cf. Cortese & Fugett, 2004).

For *emotionality* ratings, participants were instructed to rate the nouns regarding their capacity to elicit pleasant or unpleasant and awkward feelings or emotions (cf. Rubin & Friendly, 1986) on a scale from −100 to 100. Specifically, they were instructed to assign high positive numeric values if the presented word elicited positive and pleasant feelings or emotions, and to assign large negative numeric values if the presented word elicited negative and unpleasant feelings or emotions; neutral words that elicited neither positive nor negative feelings or emotions should be assigned a value close to zero. Moreover, participants were also explicitly advised only to rate the presented word and not to include associated words that were evoked by the presented word in their rating. The specific wording of the instructions for imageability and emotionality ratings is provided in the Appendix.

## Results

### Reliability of ratings

**Fig. 3:**
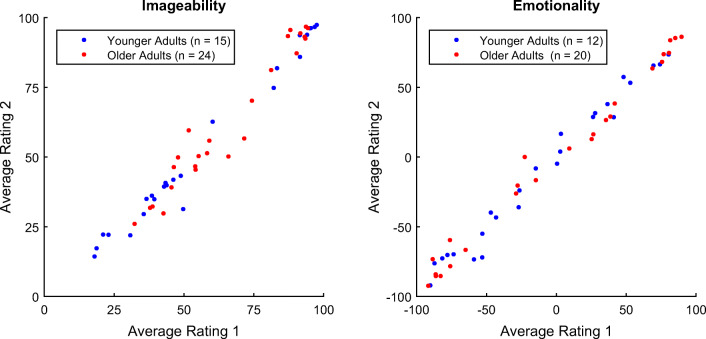
Reliability of the average imageability and emotionality ratings. Dots represent the 25 anchor words, and Ratings 1 and 2 refer to the word ratings obtained from two sessions.

The internal consistency of ratings by different participants for the 25 anchor items, as assessed with Cronbach’s *α*, was high for both age groups and for the imageability as well as for the emotionality ratings (*α* ≥ .97; Table 1). Similarly, across sessions, the rank-order stability of the average imageability and emotionality ratings for the 25 anchor words was high within both age groups (*r* ≥ .97; Fig. 3, Table 2). However, it should be noted that *α* and reliability was estimated only for the 25 anchor words presented at the very beginning of the rating session; as a consequence, the consistency and stability values presented here may not be representative of the full set of words.

**Table 1:** Cronbach’s *α* for the ratings of the first 25 words in the first session, rated by all participants

Age Group	Imageability		Emotionality	
	*n*	*α*	*n*	*α*
Young	29	.99	26	.99
Old	32	.97	25	.99

*n* = number of participants; *α* = Cronbach’s alpha assessing the internal consistency of ratings by different participants

**Table 2:** Retest stability of the aggregated imageability and emotionality ratings across two sessions (25 words)

Age Group	Imageability			Emotionality		
	*n*	*r*	*ρ*	*n*	*r*	*ρ*
Young	15	.99	.97	12	.99	.99
Old	24	.97	.94	20	.99	.99

*n* = number of participants contributing to the aggregated ratings; *r* = Pearson correlation coefficient; *ρ* = Spearman correlation coefficient

### Validity of the ratings

**Fig. 4:**
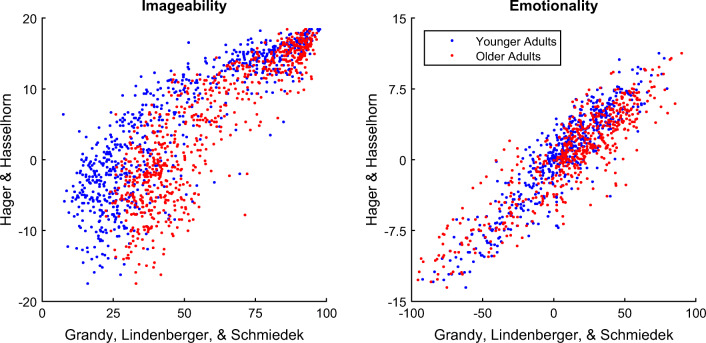
Validity of the ratings in our study as compared to the word norms reported in Hager and Hasselhorn (1994b).

The correlations with the word norms previously reported in Hager and Hasselhorn (1994b) for imageability (*Bildhaftigkeit*) and emotionality (valence; *Valenz*) were very high (*r* ≥ .87; Fig. 4, Table 3), indicating the validity of our procedure and ratings as compared to the existing German word norms. Moreover, no age differences could be observed with respect to the rank-order stability of imageability and emotionality ratings as compared to the older word norms (i.e., equally high correlation coefficients across both age groups).

**Table 3:** Validity of the ratings, as compared to the word norms reported in Hager and Hasselhorn (1994b)

Age Group	Imageability (725 words)		Emotionality (556 words)	
	*r*	*ρ*	*r*	*ρ*
Younger	.87	.86	.92	.90
Older	.87	.86	.89	.86
Younger and older	.88	.87	.93	.91

*r* = Pearson correlation coefficient; *ρ* = Spearman correlation coefficient

### Mean level differences of ratings and correlations of the ratings between age groups

**Fig. 5:**
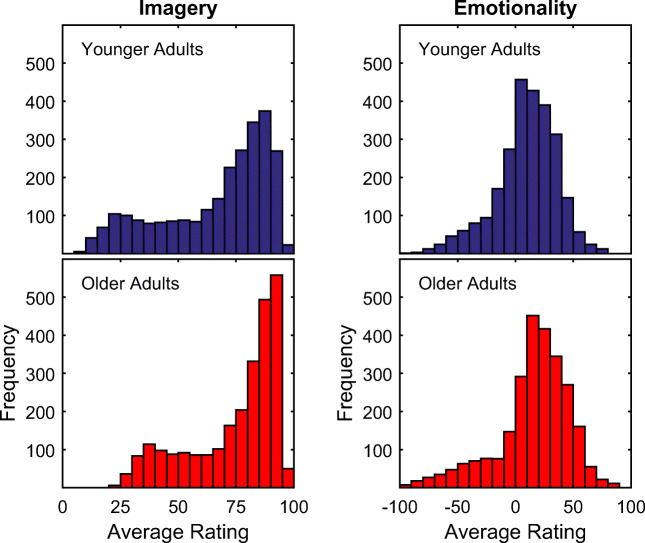
Age comparison of the distributions of ratings for imageability and emotionality.

Absolute differences in the overall mean levels of ratings between the age groups were observed, whereas no clear effect of gender was found (Table 4). The mean level difference amounted to eight points on the 0 to 100 scale (Cohen’s *d* = 0.38) for imageability, and six points on the −100 to 100 scale (*d* = 0.20) for emotionality ratings, with a higher mean level for older adults on both rated categories. Exhaustion of the rating scale was higher for younger adults for the imageability ratings, with older adults making on aggregate less use of the lowest 20% of the scale, explaining the age difference in imageability ratings. For emotionality ratings, older adults used the scale more extensively than did younger adults, with a shift toward more positive emotionality ratings (Fig. 5).

**Table 4:** Age and gender differences in the mean levels of ratings

Age Group	Gender	Imageability		Emotionality	
		Mean ± *SD* [Range]	*d*	Mean ± *SD* [Range]	*d*
Young	Male	66.3 ± 21.9 [6.7 97.1]	0.02	9.3 ± 26.0 [− 87.2 80.1]	0.02
	Female	66.7 ± 25.9 [4.2 98.6]		9.8 ± 28.4 [− 97.4 85.9]	
Old	Male	74.8 ± 21.4 [13.3 98.9]	– 0.03	15.4 ± 32.1 [− 100.0 92.8]	0.00
	Female	74.3 ± 17.8 [20.3 96.9]		15.3 ± 32.1 [− 96.9 90.4]	
Young		66.5 ± 23.5 [7.4 97.7]	0.38	9.5 ± 26.3 [− 92.3 79.9]	0.20
Old		74.6 ± 19.3 [22.4 97.1]		15.4 ± 31.4 [− 98.4 90.0]	

*d* = Cohen’s *d*

**Fig. 6:**
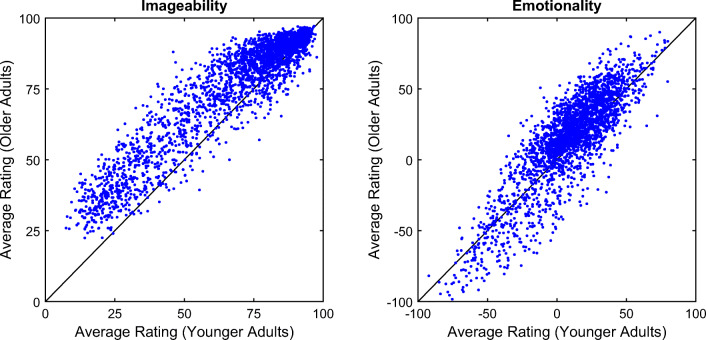
Correlations of average ratings across age groups. The black lines indicate where identical ratings between younger and older adults would lie and help visualize differences in the ratings between younger and older adults.

Correlations of the average ratings across gender and age groups were very high (Table 5). Within age groups, the correlations between males and females were at least *r* = .87. Across age groups, the correlations were found to be *r* = .94 for imageability and *r* = .85 for emotionality ratings (Fig. 6).

**Table 5:** Correlations of average ratings across subgroups

Groups	Imageability		Emotionality	
	*r*	*ρ*	*r*	*ρ*
Young (Female vs. Male)	.94	.91	.87	.84
Old (Female vs. Male)	.91	.85	.91	.86
Young vs. Old (Female and Male)	.94	.89	.85	.81

*r* = Pearson correlation coefficient; *ρ* = Spearman correlation coefficient

### Characteristic differences in word ratings between age groups

A closer look at the words with the largest age differences in imageability and emotionality revealed characteristic differences between the age groups (Table 6), underscoring the usefulness of age-specific norms. Furthermore, younger adults indicated words more often as “unknown.” Across the 2592 words presented, 29 words for younger and only three words for older adults were indicated in more than 5% of the ratings as “unknown.” In all, 2471 words (95.3%) received valid (i.e., no “unknown”) ratings by all participants. Table 7 provides an overview of the most frequent words indicated as “unknown.”

**Table 6:** Largest differences in average ratings between older (OA) and younger (YA) adults

Imageability							
Ratings YA > OA				Ratings YA < OA			
Word	Δ	YA	OA	Word	Δ	YA	OA
Fee (fairy)	20	77	57	Sohn (son)	– 42	46	88
Gespenst (ghost)	20	85	65	Garbe (sheaf)	– 37	47	84
Pluto (Pluto)	16	66	50	Rückgrat (backbone)	– 36	40	77
Schimmer (glimmer)	16	55	39	Schlemmer (glutton)	– 36	33	68
Mammut (mammoth)	15	91	76	Sonntag (Sunday)	– 35	41	76
Schleim (slime)	14	79	65	Gramm (gram)	– 35	26	61
Hexe (witch)	14	87	73	Rundfunk (broadcast)	– 34	38	72
Teufel (devil)	14	80	66	Gehalt (salary)	– 34	29	63
Pinzette (tweezers)	14	95	81	Waise (orphan)	– 34	35	69
Stinktier (skunk)	14	87	73	Rätsel (puzzle)	– 33	42	75
Emotionality							
Ratings YA > OA				Ratings YA < OA			
Word	Δ	YA	OA	Word	Δ	YA	OA
Panther (panther)	58	36	− 23	Disziplin (discipline)	– 63	12	51
Gewitter (tempest)	53	22	− 31	Arbeit (work)	– 53	− 9	44
Vampir (vampire)	53	− 4	− 57	Pflicht (duty)	− 52	− 26	26
Unterwelt (underworld)	51	− 14	− 66	Papst (pope)	− 52	− 35	17
Dschungel (jungle)	51	30	− 21	Gott (god)	− 50	2	52
Bumerang (boomerang)	49	37	− 12	Glucke (clucking hen)	− 50	− 22	28
Drache (dragon)	49	17	− 32	Elite (elite)	− 49	− 18	31
Revolver (revolver)	49	− 26	− 75	Wohnblock (housing block)	− 48	− 24	24
Floh (flea)	49	− 17	− 66	Pfleger (nurse)	− 48	− 7	41
Gespenst (ghost)	48	− 3	− 51	Kapelle (chapel)	− 45	9	54

Δ = age difference in ratings

**Table 7:** Most frequent “unknown” words by younger and older adults

Younger Adults		Older Adults	
Word	%	Word	%
Garbe (sheaf)	32	Tunika (tunic)	11
Egge (harrow)	25	Kaftan (caftan)	7
Zobel (sable)	24	Amphore (amphora)	7
Litanei (litany)	22		
Kaftan (caftan)	21		
Quaste (tassel)	21		
Schalmei (shawm)	21		
Blesse (blaze)	19		
Neglige (negligee)	18		
Humpen (beaker)	17		

% = relative frequency of participants indicating the word as “unknown” (across imageability and emotionality ratings)

## Discussion

The goal of the present study was to collect age-comparative imageability and emotionality ratings for a large body of German nouns from younger (21–31 years) and older (70–86 years) adults, and to make this information available to other researchers. Ratings were initially collected to control for imageability and emotionality of words across two age groups in memory tasks in a large-scale cognitive training study (Schmiedek et al., 2010) as these word characteristics have been repeatedly reported to influence recognition and recall probability of words (e.g., Adelman & Estes, 2013; Cortese, McCarty, & Schock, 2010; Cortese et al., 2015; Kensinger et al., 2002; Paivio et al., 1969; Rubin & Friendly, 1986). Nonetheless, the information may prove useful also in other experimental contexts (e.g., Balota et al., 2004; Cortese & Fugett, 2004).

Internal consistency (Cronbach’s *α*) and retest rank-order stability of ratings were very high for both age groups. Also, the validity of our ratings as compared to ratings collected and published by Hager and Hasselhorn (1994b) was found to be very high (*r* ≥ .86). Thus, our ratings provide a reliable and valid representation of the constructs imageability and emotionality (valence) as compared to previous instantiations, which ensures that findings obtained with our ratings may be integrated into the existing literature on a sound basis.

The ratings showed substantial rank-order stability across younger and older adults (imageability, *r* = .94; emotionality, *r* = .85). That is, younger and older adults displayed on a general level a large degree of agreement regarding imageability and emotionality of nouns. Nevertheless, younger and older adults differed in the overall mean levels of ratings (imageability, *d* = 0.38; emotionality, *d* = 0.20) as well as the extent to which they used the rating scales (imageability, *SD* = 24 vs. 19; emotionality, *SD* = 26 vs. 31), indicating a slightly higher mean anchor for the scales in the older adults, with a less extant use of the rating scale for imageability and a more extant use of the rating scale for emotionality. Figure 6 reveals that for imageability older adults rated words consistently higher than younger adults, whereas for emotionality, older adults rated words of positive emotionality more positive and of negative emotionality more negative than younger adults (in line with Grühn & Smith, 2008). Accordingly, for imageability the mean level difference was more pronounced between age groups, whereas the usage of the rating scale was reduced in the older adults due to a “bias” toward higher imageability. A likely explanation for this finding is that older adults due to their larger cumulative experience across their lifespan more easily invoke a mental image for a word than younger adults.

In a similar vein, characteristic differences in imageability and emotionality ratings were observed between younger and older adults (Table 6). For instance, for older adults the better imageability of the word “son” quite plausibly results from concrete life experiences they can resort to which younger adults have not (yet) made. On the other hand, younger adults may have had more exposition, for instance, to visual media (movies) that contain mythical or fantasy creatures like “fairies,” “ghosts,” “witches,” and so forth, and as a consequence more easily invoke mental images for these words than older adults do. Similarly, the distinctly more positive emotionality ratings of “discipline”, “work”, “duty”, “pope”, “god”, or “chapel” of older adults may be strongly reflecting generational and societal changes. Notably, as Table 6 shows the words with the largest rating differences between age groups, it appears that differences between age groups may be rooted in different evaluations of whole semantic categories, as, for instance, the abovementioned words may be subsumed under semantic categories like “myths/fantasy,” “religion,” or “virtues.” Furthermore, in line with the notion of higher crystallized intelligence (“pragmatics”) as a consequence of lifelong acquisition of knowledge (Baltes, 1987; Baltes, Staudinger, & Lindenberger, 1999), older adults indicated far fewer words as “unknown” than younger adults. In sum, and importantly so, our findings indicate that there are systematic differences between younger and older adults and strongly support our conjecture that the existence of age-specific imageability and emotionality norms is highly desirable for research on cognitive aging and the cognitive neuroscience of aging.

To exemplify this point, in a comprehensive study by Balota et al. (2004) imageability has been shown to explain a substantial amount of variance in reaction times in a lexical decision task for both younger and older adults over and above several phonological features and lexical variables. In addition, an age dependent effect was observed in the amount of additional variance explained; on the basis of the Cortese and Fugett (2004) imageability ratings (obtained from undergraduate students), imageability accounted for an additional 7.2% of variance in reaction times in younger adults, as compared to 5.3% in older adults. Although this may be attributable to age-related differences in cognitive processes engaged (e.g., older adults relying less on semantic information in this specific task), an alternative explanation is that the lower additional variance explained in older adults is due to a limited generalizability of the imageability norms obtained from younger adults to older adults. That is, because we observed systematic differences in the imageability of words between younger and older adults, variance in the imageability of words in older adults was not accounted for by the imageability ratings of younger adults, thus reducing their predictive power for older adults. Of note, this is not to say that younger adults’ ratings are to be considered generally invalid for predicting the cognitive performance of older adults. In line with the high rank-order stability between the ratings of younger and older adults observed in our study, the general correlational pattern in the Balota et al. (2004) study was found to be consistent across age groups. Nevertheless, this example well illustrates that when dissecting more subtle differences between age groups, as in this case the differential utilization of semantic variables in lexical decision, it may prove difficult to fully distinguish between true age-related differences in actual cognitive processing and age-related differences induced by word ratings that are biased to some degree, when these were obtained from only one age group.

To conclude, the age-specific imageability and emotionality ratings for 2592 German nouns collected in this study provide useful information to control or manipulate stimulus material in experiments that involve younger and older adults. Previous findings highlighted the significance of imageability and emotionality of words, for instance, for their memorability. The existence of considerable and characteristic differences between the two age groups underscores the importance of carefully matching word material across age groups. The word ratings can be found in the electronic supplementary material 2.

### Author note

We thank Colin Bauer for programming the task, as well as all research assistants involved in data collection. We thank Michael Cortese for a kind and helpful review.

## Electronic supplementary material

ESM 1 (DAT 460 kb)

ESM 2 (DAT 2.60 kb)

ESM 3 (DOCX 49.2 kb)
