# Supplementary material for: Vampires and nurses are rated differently by younger and older adults—Age-comparative norms of imageability and emotionality for about 2500 German nouns
Source: Behav Res Methods. 2020 Feb 12;52(3):980–9. doi: 10.3758/s13428-019-01294-2 (PMC7280351; doi:10.3758/s13428-019-01294-2)
Supplement: Supplementary file 3 — (DOCX 49.2 kb) [file 13428_2019_1294_MOESM3_ESM.docx]

# Appendix

## Instruction – Imageability (German)

| Wörter unterscheiden sich in ihrer Eigenschaft, **bildhafte Vorstellungen von Dingen, Ereignissen oder Geräuschen hervorzurufen**. Einige Wörter bewirken sehr schnell und leicht das Auftreten einer bildhaften Vorstellung, eines Ereignisses oder eines Klangs. Andere Wörter tun dies nur sehr schwer und langsam oder überhaupt nicht.  (Im Folgenden wird nur noch von bildhafter Vorstellung die Rede sein. Gleichzeitig ist aber immer auch die Vorstellung eines Ereignisses oder Klangs gemeint.)  In dieser Studie sollen Sie insgesamt um die 2.500 Substantive danach beurteilen, wie leicht oder schwer sie bildhafte Vorstellungen hervorrufen.  Die Bewertung wird auf einer **Skala von 0 bis 100** erfolgen.   - Jedes Wort, das eher schnell und leicht eine bildhafte Vorstellung auslöst, sollte einen hohen Zahlenwert erhalten. - Jedes Wort, das bei Ihnen nur schwer und langsam oder aber überhaupt keine bildhafte Vorstellung erzeugt, sollte einen niedrigen Zahlenwert erhalten. - Wörter, die Ihrer Meinung nach weder leicht noch schwer bildhafte Vorstellungen auslösen, sollten mit einem Zahlenwert um 50 bewertet werden.   Betrachten wir z.B. die Wörter „Genauigkeit“ und „Teebeutel“. Das Wort „Genauigkeit“ wird vermutlich eher langsam eine bildhafte Vorstellung auslösen und sollte daher einen entsprechend niedrigen Wert erhalten. Das Wort „Teebeutel“ hingegen wird eher schnell eine bildhafte Vorstellung wecken und sollte daher entsprechend hoch bewertet werden.  Wörter können die Vorstellung anderer Wörter hervorrufen: „Teebeutel“ z.B. „Kanne“ oder „Tasse“. Es ist sehr wichtig, dass Sie **ausschließlich das jeweils vorgegebene Wort beurteilen** und andere Wörter dabei nicht berücksichtigen.  **Beachten Sie bei der Bewertung bitte folgende Dinge:**   1. Bei persönlichen Urteilen gibt es keine „richtigen“ oder „falschen“ Antworten! 2. Arbeiten Sie zügig, aber sorgfältig. 3. Bewerten Sie bitte *jedes* Wort. 4. Machen Sie sich keine Gedanken darüber, wie oft Sie eine bestimmte Zahl ausgewählt haben, so lange die Zahlen Ihr persönliches Urteil widerspiegeln. 5. Versuchen Sie alle Wörter nach dem gleichen Standard zu bewerten. Hierfür sind zu Ihrer Orientierung die ersten 25 Wörter so ausgewählt, dass sie etwa das gesamte Spektrum der Bildhaftigkeit repräsentieren. Eine Liste dieser 25 Wörter finden Sie auch an Ihrem Arbeitsplatz. Diese Liste können Sie im Laufe der Bewertung immer wieder zu Hilfe nehmen, um einen einheitlichen Bewertungsstandard beizubehalten.   **Wenn Sie während der Bewertung Fragen haben sollten, lesen Sie sich bitte die Instruktion noch einmal durch.** |
| --- |

## Instruction – Imageability (English translation)

| Words differ in their capacity to **elicit imaginations of objects, events, or sounds**. Some words effectuate the appearance of an imagination of an object, event, or sound easily and fast. Other words do so only with difficulty, slowly, or not at all.  (In the following we will refer to ‘mental images’ while also referring to the imagination of events, or sounds.)  In this study, we ask you to rate about 2,500 nouns regarding how easily or difficultly they evoke mental images.  Your ratings will be made on a **0 to 100** scale.   - Words evoking easily and fast a mental image should be assigned high numerical values. - Words evoking a mental image only with difficulty, slowly, or not at all should be assigned low numerical values. - Words that in your opinion evoke a mental image neither easily nor with difficulty should be assigned numerical values around 50.   Let’s consider, for instance, the words ‘precision’ and ‘tea bag‘. The word ‘precision’ will presumably only slowly elicit a mental image and should therefore be assigned a small numerical value. The word ‘tea bag’ on the other hand will likely evoke a mental image rather fast and should therefore be assigned a high numerical value.  Words can elicit mental images of other words. For example, ‘tea bag’ may elicit the words ‘tea pot’ or ‘cup’. It is very important that you **rate exclusively the actual presented word** and do not consider other words in your rating.  **Please consider the following points when rating the words:**   1. There are no ‘right‘ or ‘wrong‘ answers when providing personal ratings! 2. Do not spend too much time on any word, but try to be as accurate as possible at the same time. 3. Please rate *every* word. 4. Don’t consider how often you have chosen a specific numerical value, as long as the numbers reflect your personal judgement. 5. Try to rate all words with the same standard. For your orientation, the first 25 words are chosen to represent approximately the full spectrum of imageability. A list of these 25 words is also provided on your desk. You can resort to this list during the rating at all times in order to ensure a consistent standard.   **Please consult this instruction again if you have any questions during the rating procedure.** |
| --- |

## Instruction – Emotionality (German)

| Wörter können in unterschiedlichem Maße **angenehme oder unangenehme Gefühle** **bzw. Emotionen** **auslösen**. Im Folgenden sollen Sie daher insgesamt um die 2.500 Substantive danach beurteilen, ob diese eher ein angenehmes oder ein unangenehmes Gefühl hervorrufen.  Die Bewertung wird auf einer **Skala von –100 bis +100** erfolgen.   - Jedes Wort, das ein positives, angenehmes Gefühl in Ihnen weckt, sollte einen hohen positiven Zahlenwert erhalten. - Jedes Wort, das ein negatives, unangenehmes Gefühl bei Ihnen erzeugt, einen hohen negativen Zahlenwert erhalten. - Neutrale Wörter, die also Ihrer Meinung nach kaum positive oder negative Gefühle auslösen, sollten mit einem Zahlenwert nahe 0 bewertet werden.   Betrachten wir z.B. die Wörter „Gewalt“ und „Freude“. Das Wort „Gewalt“ wird vermutlich ein unangenehmes Gefühl hervorrufen und sollte daher einen entsprechend negativen Wert erhalten. Das Wort „Freude“ hingegen wird eher ein angenehmes Gefühl wecken und sollte daher entsprechend positiv bewertet werden.  Wörter können die Vorstellung anderer Wörter hervorrufen: „Freude“ z.B. „Spaß“ oder „Stimmung“. Es ist sehr wichtig, dass Sie **ausschließlich das jeweils, vorgegebene Wort beurteilen** und andere Wörter dabei nicht berücksichtigen.  **Beachten Sie bei der Bewertung bitte folgende Dinge:**   1. Bei persönlichen Urteilen gibt es keine „richtigen“ oder „falschen“ Antworten! 2. Arbeiten Sie zügig, aber sorgfältig. 3. Bewerten Sie bitte *jedes* Wort. 4. Machen Sie sich keine Gedanken darüber, wie oft Sie eine bestimmte Zahl ausgewählt haben, solange die Zahlen Ihr persönliches Urteil widerspiegeln. 5. Versuchen Sie alle Wörter nach dem gleichen Standard zu bewerten. Zu Ihrer Orientierung sind daher die ersten 25 Wörter so ausgewählt, dass sie etwa das gesamte Spektrum des Emotionsgehaltes repräsentieren. Eine Liste dieser 25 Wörter finden Sie auch an Ihrem Arbeitsplatz. Diese Liste können Sie im Laufe der Bewertung immer wieder zu Hilfe nehmen, um einen einheitlichen Bewertungsstandard beizubehalten. 6. Es kann vorkommen, dass ein Wort in Ihnen widersprüchliche, d.h. positive und negative Gefühle gleichzeitig, auslöst. In einem solchen Fall tun Sie bitte Folgendes: Entscheiden Sie, ob die positiven oder die negativen Gefühle überwiegen. Bewerten Sie anschließend das Wort so, als hätte es nur die überwiegende Emotion ausgelöst. Bilden Sie bitte keine „Kompromisslösung“, die die Stärke der positiven und negativen Gefühle verbindet.   **Wenn Sie während der Bewertung Fragen haben sollten, lesen Sie sich bitte die Instruktion noch einmal durch.** |
| --- |

## Instruction – Emotionality (English translation)

| Words differ in their capacity to **arouse** **pleasant or unpleasant feelings or emotions**. In the following, we ask you to rate about 2,500 nouns regarding their capacity to evoke pleasant or unpleasant feelings.  Your ratings will be made on a **–100 to +100** scale.   - Words evoking positive and pleasant feelings or emotions should be assigned high positive numerical values. - Words evoking negative and unpleasant feelings or emotions should be assigned high negative numerical values. - Words that evoke little positive or negative feelings or emotions should be assigned numerical values close to 0.   Let’s consider, for instance, the words ‘violence’ and ‘joy‘. The word ‘violence’ will presumably elicit an unpleasant feeling or emotion and should therefore be assigned a corresponding negative numerical value. The word ‘joy’ on the other hand will rather evoke a pleasant feeling or emotion and should therefore be assigned a corresponding positive numerical value.  Words can elicit the imagination of other words. For example, ‘joy’ can elicit the words ‘fun’ or ‘spirit’. It is very important that you **rate exclusively the actual presented word** and do not consider other words in your rating.  **Please consider the following points when rating the words:**   1. There are no ‘right‘ or ‘wrong‘ answers when providing personal ratings! 2. Do not spend too much time on any word, but also try to be as accurate as possible. 3. Please rate *every* word. 4. Don’t consider how often you have chosen a specific numerical value, as long as the numbers reflect your personal judgement. 5. Try to rate all words with the same standard. For your orientation, the first 25 words are chosen to represent approximately the full spectrum of emotionality. A list of these 25 words is also provided on your desk. You can resort to this list during the rating at all times in order to ensure a consistent standard. 6. It is possible that words elicit conflicting, that is, positive and negative feelings and emotions at the same time. In this case, please proceed as follows: Decide whether positive or negative feelings prevail; then rate the word as if it had only elicited the prevailing feeling or emotion. Please do not compose some ‘compromise’ that combines the strength of the positive and negative feelings and emotions.   **Please consult this instruction again if you have any questions during the rating procedure.** |
| --- |

## Anchor List – Imageability

Following 25 words were presented first in each session (in random order) in order to ensure comparable anchoring of imageability ratings across participants:

Jahr (year), Abruf (recall), Mittel (means), Geschichte (story), Ende (end), Welt (world), Ruf (call), Grund (ground), Belang (concern), Uhr (clock), Erdbeere (strawberry), Frevel (sacrilege), Frage (question), Stuhl (chair), Sorge (worry), Kind (child), Verzug (delay), Zeit (time), Frosch (frog), Kreis (circle), Hammer (hammer), Ziel (goal), Sinn (meaning), Bild (picture), Staat (state).

## Anchor List – Emotionality

Following 25 words were presented first in each session (in random order) in order to ensure comparable anchoring of emotionality ratings across participants:

Gefängnis (prison), Mädchen (girl), Fall (case), Frage (question), Liebe (love), Bild (picture), Gesundheit (health), Kuss (kiss), Kind (child), Forderung (claim), Macht (might), Uhr (clock), Zeit (time), Grund (ground), Bedrohung (threat), Angriff (attack), Freund (friend), Krankheit (disease), Heiterkeit (cheerfulness), Problem (problem), Welt (world), Mord (murder), Ausrottung (eradication), Angst (anxiety), Tod (death).
